# Explaining the negative effects of workplace incivility on family lives: a moderated mediation model of surface acting and resource-providing variables

**DOI:** 10.3389/fpsyg.2024.1409144

**Published:** 2024-07-16

**Authors:** Yuanbo Gu, Cuiping Wang, Jinhua Ma

**Affiliations:** Luoyang Normal University, Luoyang, Henan Province, China

**Keywords:** workplace incivility, surface acting, supervisor work–family support, psychological detachment, work-to-family interference

## Abstract

The effects of workplace incivility have been understudied in educational settings. To expand incivility research to educational professions, the present research investigates whether, how, and when workplace incivility deriving from different sources (coworkers, supervisors, and outsiders) is related to work-to-family interference (WFI) of preschool teachers. Drawing on the conservation of resources theory, the present study proposes that workplace incivility and subsequent maladaptive emotion labor strategies (i.e., surface acting) jointly create a resource-depletion mechanism contributing to elevated WFI and two resource-providing variables (supervisor work–family support and psychological detachment after hours) function as potential mitigating factors to break the resource-depletion mechanism. This study used a female-dominated sample (i.e., preschool teachers) found that workplace incivility from insiders (supervisors and coworkers respectively) and external stakeholders (child’s family members) all positively linked to WFI, and surface acting mediated these relationships. Moreover, moderated mediation analyses indicated that psychological detachment buffered the mediated effect of surface acting on WFI, whereas supervisor work–family support did not. Findings deepen the understanding of why and when workplace incivility influences employees’ family lives, as well as point to future intervention strategies.

## Introduction

In recent years, interest in workplace mistreatment and its destructive influence has risen sharply. Workplace mistreatment including conflict, incivility, bullying, and aggression are considered as one of the most frustrating stressors in the workplace, posing a potential threat to relatedness, self-confidence, and social companionship, and ultimately leading to serious and deleterious influences on employee health, wellbeing and job performance (Almeida, 2005; Lim et al., 2008; Chi et al., 2016; Schilpzand et al., 2016; Baranik et al., 2017; Tarrafa et al., 2019; He et al., 2021). Workplace incivility (*hereafter incivility*) is a form of mistreatment that refers to “a low-intensity deviant behavior with ambiguous intent to harm the target, in violation of workplace norms for mutual respect” (Andersson and Pearson, 1999, p. 457). Incivility has been classically depicted as “treatment that is discourteous, rude, impatient, or otherwise showing a lack of respect or consideration for another’s dignity” (Kane and Montgomery, 1998, p. 266). Such incivility includes disrespectful and/or derogatory remarks, snippy e-mails, gossip, talking down to others, being frequently interrupted, ignoring someone, and making demeaning comments about others (Lim and Lee, 2011; Cortina et al., 2017).

Being different from other types of workplace mistreatment, such as aggression, workplace bullying and abusive supervision, incivility represents a milder form of mistreatment with three features–low intensity, unclear intention, and rule violation (Andersson and Pearson, 1999; Cortina et al., 2022). Low intensity means actors not displaying severe deviant behavior such as aggressive or physical acts, and as such some researchers argue that incivility represents a lower end of the severity or intensity continuum and it is likely to escalate into more serious conflict and aggression (Nguyen and Stinglhamber, 2020; Yao et al., 2022; Freedman et al., 2024). Unclear intention means actors not carrying a conscious intent to psychologically or physically harm others, so incivility can also be considered as an implicit form of mistreatment (Sliter et al., 2014; Cortina et al., 2022; Martin and Zadinsky, 2022). Rule violation means actors not following the principles of interpersonal interaction, and some scholars view incivility as a subset of counterproductive work behavior or the opposite of organizational citizenship behavior (Cortina et al., 2017; Park and Martinez, 2022; Lages et al., 2023).

The voluminous evidence has indicated that employees commonly report lower job satisfaction (Cortina et al., 2001; Hershcovis et al., 2017), heightened burnout (Sliter et al., 2010), diminishing job performance (Caza and Cortina, 2007), embarrassment and perceived job insecurity (Hershcovis et al., 2017), insomnia symptoms (Demsky et al., 2019), increased depression (Lim and Lee, 2011), and decreased physical and psychological health (Lim et al., 2008) after experiencing a high degree of incivility. Consequences of incivility can also go beyond work boundaries. A handful of studies have indicated that incivility has a robust relationship with lower marital satisfaction as well as higher work–family interference (e.g., Lim and Lee, 2011; Ferguson, 2012; Zhou et al., 2019).

Although the damaging effects of incivility has garnered considerable empirical support, the underlying mechanisms regarding why and when incivility leads to these effects has been largely overlooked. Drawing on the conservation of resources (COR) theory (Hobfoll, 1989), this study strives to (a) test the comparative effect that incivility from different sources (i.e., supervisors, coworkers, and family members) can have on employees’ personal lives (i.e., work-to-family interference; WFI); (b) identify a potential mechanism (i.e., surface acting) by which the relationship between incivility and WFI can be partially and completely explained; and (c) deepen the understanding of the boundary conditions of incivility by identifying supervisor work–family support and psychological detachment as resource-providing variables that mitigate the adverse effects of workplace incivility. Overall, based on COR theory, we developed and empirically tested a moderated mediation model, in which surface acting mediates the incivility–WFI relationship and both supervisor work–family support and psychological detachment buffer the surface acting–WFI relationship. An integrated moderated mediation can advance our understanding of “how” and “when” incivility impacts the quality of employees’ family lives. We tested our hypotheses in a female-dominated occupational field: preschool teachers in early childhood education, a profession largely overlooked by incivility researchers. Research spanning various fields uniformly indicates women are especially likely to experience interpersonal mistreatment in the workplace (Chan et al., 2008; Lim and Lee, 2011; Sliter et al., 2014). By doing so, the contributions of this research are as follows:

First, researchers suggested the source from which incivility is perceived (e.g., outsiders, supervisors, or coworkers), is worth exploring because the impacts of incivility may rely on these sources (Grandey et al., 2007; Cortina and Magley, 2009; Lim and Lee, 2011; Ferguson, 2012; Demsky et al., 2019). Based on the information we have so far, no research to date has identified the relative influence that incivility from different sources can have on preschool teachers. In the early childhood education settings, incivility can mostly instigate by supervisors and coworkers, as in most professions. To illustrate the benefits of occupation-specific incivility research, we also examined a unique source of incivility to preschool teachers, namely incivility stemming from child’s family members. This study aims to find the impact caused by incivility originating from the three stakeholders of the preschool teachers’ role to preschool teachers. In this study, we differentiate between three sources of incivility (i.e., coworkers, supervisors, and child’s family members) and test their relations to WFI simultaneously. These three sources of incivility are investigated in the same research to help provide a more holistic picture regarding the unique as well as differential influences of incivility from multiple sources for preschool teachers.

Second, because work and family are becoming increasingly antagonistic (Kinnunen et al., 2016; Kubicek and Tement, 2016), understanding the underlying mechanism that drive WFI is of imperative significance in balancing both work and family roles. The present research integrates emotional labor into the incivility literature to better understand the relationship between incivility and WFI. That is, except for the direct impacts of incivility on WFI, incivility can also exert an indirect impact on WFI via emotional labor. Because incivility is emotion-provoking events arising from interactions with others, emotional labor, center on the modification of emotional expressions in order to comply with organizationally prescribed emotions during interpersonal interactions (Hochschild, 1983; Grandey, 2000), might be a primary coping response by which this relationship between incivility and WFI might be understood. Previous studies have yet to elucidate the role of emotional labor in explaining the potentially adverse effect of incivility on employees’ family lives.

Lastly, this research contributes to the existing literature by strengthening the significance of identifying personal and situational moderators that buffer the mediated effect of surface acting on WFI. Given the emotional challenges in the early childhood education settings, we conceptualize two resource-providing variables (i.e., supervisor work–family support and psychology detachment) as the two remarkable stress buffers that are proposed to prevent the secondary resource losses of surface acting due to incivility. We introduce supervisor work–family support and detachment as the two malleable resource-providing variables that have been shown to have important intervention implications (Hahn et al., 2011; Kossek et al., 2011; Karabinski et al., 2021).

## Theoretical background: the conservation of resources theory

The COR theory (Hobfoll, 1989) is a stress and motivational theory that underscores the critical role of resource loss, resource gain, and resource lack and possession. COR theory starts with the tenet that people seek to protect, replenish, acquire, and conserve various resources (Hobfoll, 1989, 2002), such as conditions (e.g., promotion), objects (e.g., food), personal characteristics (e.g., learned skills), and energies (e.g., money). Psychological strain takes places or develops when individuals experience a threat of resource loss, an actual of resources, or a lack of resource gain after significant effort. Thus, there is reason to suspect that incivility tends to result in a loss of resources, such as social identity, good social relations, and favorable self-evaluation, and reduces the number available to family role (Edwards and Rothbard, 2000). Thus, resource loss can be seen as a key reason for interpreting and predicting the occurrence of the incivility–WFI relationship.

The another tenet of COR theory is that initial resource loss can beget further loss, and this occurs when a stressor evokes a maladaptive coping response (Hobfoll, 2002; Ten Brummelhuis and Bakker, 2012). Incivility as an emotion-provoking event either threatens the loss or entails the actual loss of affective resources such as positive feelings about oneself and intimacy and affection from others, and cognitive resources such as attentional resources and social identity (Zhou et al., 2015). Employees who experience incivility will have to make an extra effort to perform surface acting, which further requires consume, and thus loss, of resources.

Finally, COR theory assumes that resources can be used to handle stressful circumstances and that individuals who possess abundant resources are more likely to avoid problematic situations (Hobfoll, 2002). Given that both supervisor work–family support and detachment can be seen as resource-providing variables through which individuals can preserve and produce other valued resources (Hobfoll, 2002), presently, we propose that they can help employees buffer against the detrimental effects of incivility and subsequent surface acting on employees’ family lives. Below, we will utilize the COR theory as a pivotal theory to formulate hypotheses regarding the relation between incivility and WFI, as well as the mediating effect of surface acting and the buffering role of supervisor work–family support (*hereafter* SWFS) and detachment.

## Incivility affects work–family interference by creating resource depletion

Work–family interference refers to “a form of inter-role conflict in which the role pressures from the work and family domains are mutually incompatible in some respect” (Greenhaus and Beutell, 1985, p. 77). Interference between work and family lives can be bidirectional: work responsibilities interfering with family obligations (work-to-family interference; WFI) and family needs interfering with work demands (family-to-work interference; FWI) (Frone et al., 1992; Netemeyer et al., 1996; Grzywacz and Marks, 2000; Nohe et al., 2015). WFC occurs when work demands/responsibilities impede with family life, such as overtime requisites, rigid work schedules, work overload, workplace interpersonal deviance, unaccommodating coworker or supervisor, and so on (Grzywacz and Marks, 2000; Rupert et al., 2009; Demsky et al., 2014). Previous studies have consistently demonstrated that WFI is experienced more frequently than FWI by employees (e.g., Netemeyer et al., 1996; Rupert et al., 2009), and that work stressors have a stronger effect on WFI than FWI (e.g., Frone et al., 1992; Netemeyer et al., 1996) and, not surprisingly, the current study only focused on WFI.

As suggested by the COR theory, incivility increase WFI due to depletion of various energy resources. Incivility does show a robust link to resource depletion across studies. Incivility contributes to employees’ resource depletion because (a) it triggers strong negative emotions such as depression, anxiety, and frustration, which erode employees’ emotional resources (Tremmel and Sonnentag, 2017), (b) it violates organizational norms of mutual respect, which threaten or consume employees’ personal resources such as dignity, sense of value, and favorable self-evaluation (Andersson and Pearson, 1999; Porath and Pearson, 2012; Taylor et al., 2017), (c) it impedes social interactions, which threaten employees’ social-capital resources, such as social identity and good social relations (Porath and Pearson, 2012; Taylor et al., 2017), and (d) it induce incivility-related negative work rumination, which inhibits the creation of new resources during non-work time (Nicholson and Griffin, 2015; Demsky et al., 2019). Given that people’s resource pool is limited in capacity, not infinite, the resource-draining situations of incivility inevitably exhaust the total resources and reduce employees’ energy that accomplishes family tasks. Worst of all, due to excessive resource loss, individuals might try to preserve the residual resources by stop investing the limited resources to their family.

Several empirical studies have found the relationships between incivility stemming from different sources and employees’ family lives. For instance, an early research found that incivility stemming from coworkers indeed had a detrimental effect on both target and partner marital satisfaction as well as partner family-to-work interference (Ferguson, 2012). Recent research also demonstrated that incivility has a deleterious impact on employees’ non-work outcomes such as WFI and life satisfaction (He et al., 2021). Based on the empirical findings and theoretical reasoning, we propose that incivility from supervisors, coworkers, and child’s family members can all potentially expend employee resources and positively predict WFI. Meanwhile, empirical evidence does not reach a consistent conclusion about the different impacts of incivility from different sources. For example, Lim and Lee (2011) demonstrated that supervisor incivility was positively associated with WFI and incivility from subordinates and coworkers was not. Zhou et al. (2019) using an experience sampling design indicated that coworker and outsider incivility positively predicted WFI but supervisor incivility not. Research in the broader mistreatment literature hasn’t also reached a unanimous conclusion regarding the effects of mistreatment from supervisors, coworkers, and outsiders (e.g., Adams and Webster, 2013; Nguyen and Stinglhamber, 2020). Thus, we do not make differential hypotheses in our study as to the impacts of incivility from supervisors, coworkers, and family members.

Hypothesis 1: Incivility by (a) supervisors, (b) coworkers, and (c) child’s family members will positively predict WFI.

## Surface acting as a mediator in the incivility–WFI relationship

For a better understanding of the incivility–WFI relationship, we examine the possibility that this relationship is mediated by surface acting, which are often considered a key dimension of emotional labor (Hülsheger and Schewe, 2011; Grandey et al., 2012). Hochschild (1983) first introduced the concept of emotional labor, and divided it into deep acting and surface acting. Surface acting, in the form of only modifying one’s external emotional expressions by faking, suppressing, or amplifying true feelings to display “organizationally appropriate” emotions (Grandey, 2000; Grandey and Melloy, 2017), has been associated with a large quantity of harmful outcomes such as increased exhaustion, diminished job and life satisfaction as well as poor health (Brotheridge and Grandey, 2002; Hülsheger and Schewe, 2011; Grandey and Melloy, 2017). Preschool teachers are expected to continously inhibit their negative feelings (e.g., anger, anxiety, and frustration) and pretend positive emotions (e.g., happy, proud, and passionate) to accomplish pluralistic teaching goals (e.g., children’s emotional socialization, interpersonal skills, and academic success).

Based on the COR model (Hobfoll, 1989), surface acting can be considered as a maladaptive coping strategy that creates a resource-depleting process and ultimately results in harmful outcomes. Because surface acting can elicit negative emotions and result in the discrepancy between the felt and expressed emotion, individuals need to invest considerable amounts of psychological and physiological resources to monitor their actual and desired emotions constantly, and regulate their displayed emotions by faking and/or suppressing emotions (Hülsheger and Schewe, 2011; Grandey et al., 2012; Xanthopoulou et al., 2018). Accordingly, surface acting may have adverse effects on one’s nonwork domain via a loss of energy resources because resources are relatively fixed within a certain period and allocating resources to work domain reduces the investment of resources in the nonwork domain (Edwards and Rothbard, 2000). Several empirical studies have found that surface acting is closely linked to bidirectional work–family interference, especially from work to family (i.e., WFI) (Montgomery et al., 2005; Cheung and Tang, 2009; Gu and Wang, 2021). Building on the theoretical rationale and previous findings, we propose the following:

Hypothesis 2: Preschool teachers who perform more surface acting tend to experience a high level of WFI.

Surface acting was supposed to play a decisive role in dealing with workplace mistreatment (Carlson et al., 2012; Grandey et al., 2012; Adams and Webster, 2013). Employees are often required to display surface acting (e.g., faking positive emotions and/or restraining negative emotions), even in the face of cases of negative events like incivility, to maintain unwritten emotional display rules. The display rules are formed by supervisors, coworkers, and customers (Carlson et al., 2012; Adams and Webster, 2013). Incivility is accompanied by negative emotional feelings (Zhou et al., 2015; Tremmel and Sonnentag, 2017) and requires engaging in surface acting to modify the emotional response that is acceptable within their organization. In a similar fashion, preschool teachers are more likely to perform surface acting in response to incivility by suppressing negative emotions and faking positive ones.

In addition to display rules, the COR theory provides a good theoretical explanation for why incivility is more likely to result in surface acting. As mentioned above, the experience of incivility is an emotion-provoking event that would cause an employee to lose resources they value, such as positive social relationships, positive work climate, wages and welfare. Employees are motivated to seek out and maintaining these resources through suppressing or faking certain emotions (i.e., surface acting) to avoid further potential conflicts (e.g., Carlson et al., 2012). Past research revealed that employees often resort to surface acting to cope with interpersonal stressors (Carlson et al., 2012; Adams and Webster, 2013). For example, Carlson et al. (2012) showed that experiencing abusive supervision resulted mainly in the use of surface acting to cope with the abusive situation. Similarly, Adams and Webster (2013) found that employees experiencing incivility from coworkers and customers displayed surface acting to deal with the incivility. Based on the theoretical and empirical evidence, we predict:

Hypothesis 3: Preschool teachers who experience more incivility from (a) supervisors, (b) coworkers, and (c) family members are more likely to perform surface acting.

Although the pairwise relation between these three variables (incivility, surface acting, and WFI) has been demonstrated, the mediating role of surface acting in the incivility–WFI relationship has yet to be proven. As described above, employees suffering from incivility are likely to perform surface acting to follow organizational display rules and/or to avoid resource loss. However, individuals who perform more surface acting will consume their limited emotional and cognitive resources which provide them with little energy to solve family matters (Cheung and Tang, 2009). In other words, incivility is a stressor that triggers an inappropriate coping strategy—surface acting—which then consumes individuals’ resources, in turn, negatively influencing employees’ family lives. There is some empirical evidence that surface acting plays a key mediating role in the relation between other interpersonal stressors and negative work and non-work outcomes. For instance, Carlson et al. (2012) found that WFI is a function of the extent of abusive supervision and this relation is partially mediated by surface acting. Adams and Webster (2013) found that surface acting mediates the interpersonal mistreatment–psychological distress relationship. While prior research notes the mediating role of surface acting in the relationship between interpersonal mistreatment and outcomes, these earlier researches focuses on other kinds of mistreatment other than incivility, which we focus on here. The current research is the first to examine the indirect effects of various forms of incivility on WFI simultaneously through increasing surface acting by adopting a resource perspective. We believe not only that experiences of incivility is inextricably linked with surface acting and that surface acting results in WFI for preschool teachers, but that surface acting mediates the incivility-WFI relationship.

Hypothesis 4: Surface acting will mediate the relation between incivility by (a) supervisors, (b) coworkers, and (c) family members and preschool teachers’ WFI.

## The moderating influences of resource-providing variables

In addition to testing why incivility affects WFI, we also test when incivility tend to have these effects. We introduce two resource-providing variables—SWFS and detachment—as moderators of the mediated relationships between incivility and WFI via surface acting. As explained above, experiences of incivility and the subsequent surface acting response involve resource loss that would make it more difficult to perform one’s family role. It is critical to offset the resource loss and prevent future loss caused by these two interpersonal stressors. Drawing from COR theory, both resource-providing variables have the ability to boost an individual’s psychological resources, and help individuals better deal with related stress, thereby buffering against the adverse effects of incivility and subsequent surface acting on employees’ family lives.

SWFS refers to discretionary behaviors performed by supervisors that help employee fulfill family roles and meet family demands and consists of creative work-family management, role-modeling, emotional and instrumental support (Hammer et al., 2009, 2013). A supportive supervisor cares about employees’ family lives by encouraging employees to talk about family-related needs and becoming a sympathetic listener, understanding family-related requests, allowing employees to alter their work schedule, and offering assistance for the management of family issues, or demonstrating how they solve work and family problems by role-modeling (Hammer et al., 2009, 2013; Kossek et al., 2011). These create a family-friendly atmosphere in which employees are psychologically more resilient and feel more confident and respected. Indeed, empirical evidence indicates that emotional and instrumental support from a supervisor promote employees’ family and life satisfaction by minimizing work-family interference experiences (e.g., Kossek et al., 2011; Goh et al., 2015). Hence, family-supportive supervisors may boost employees’ personal resources, which, in turn, should alleviate the resource loss process that links the incivility and WFI through surface acting.

Hypothesis 5a: SWFS will buffer the surface acting–WFI relationship, such that the connection will be weaker for those who experience greater levels SWFS.

Hypothesis 5b: SWFS will weaken the strength of the mediated effects of incivility on WFI via surface acting. Specifically, the mediated effects will be weaker with higher SWFS than with lower SWFS.

Psychological detachment (*hereafter detachment*) refers to a person’s sense of separating himself or herself from work situations (Etzion et al., 1998). Detachment means not being involved in work-related tasks (e.g., not checking office e-mails) and not thinking or ruminating about work-related matters (e.g., forgetting about a work conflict with a colleague) during after-work hours and it exhibits strong, negative relations with negative affect, fatigue, and exhaustion as well as positive relations with vigor, sleep quality, physical and mental health, and life satisfaction (Sonnentag and Fritz, 2007, 2015; Bennett et al., 2018). From the COR theory perspective (Hobfoll, 2002), detachment is vital here as it provides a psychological break from work that in turn creates an opportunity to promote successful recovery from work strain by replenishing taxed resources and promoting an acquisition of energetic and affective resources (Sonnentag and Fritz, 2015; Bennett et al., 2018; Steed et al., 2019). These resources can be used to juggle work and family demands, allowing employees to reduce WFI in the face of surface acting. Detachment has been proved to play the stress buffering role in the relation between job stressors and well-being/behavior/performance including the workplace bullying–psychological strain relationship (Moreno-Jiménez et al., 2009), the job demands–well-being outcomes relationship (Sonnentag et al., 2010), the emotional conflicts–poor well-being relationship (Sonnentag et al., 2013), and the emotional dissonance–insomnia symptoms relationship (Gu et al., 2020). However, these earlier studies have focused on stressors other than socioemotional stressors, which we focus on here (i.e., incivility and emotional labor). Given theoretical reasoning and empirical evidence that engaging in detachment during non-work time can both restore threatened or lost resources as well as gain new resources (e.g., positive mood, self-efficacy), successful detachment during non-work time could to be beneficial to employees’ well-being under stressful job conditions. In the current study, we expected that detachment can offer individuals the energy to balance work and family roles and serve as a buffer of the surface acting–WFI relationship. Further, we expect detachment to buffer the mediating effects of incivility on WFI via surface acting. Hence, we propose:

Hypothesis 6a: Detachment will buffer the surface acting–WFI relationship, such that the connection will be weaker for those who report higher levels detachment.

Hypothesis 6b: Detachment will weaken the strength of the mediated effects of incivility on WFI via surface acting. Specifically, the mediated effects will be weaker with higher detachment than with lower detachment.

**FIGURE 1:**
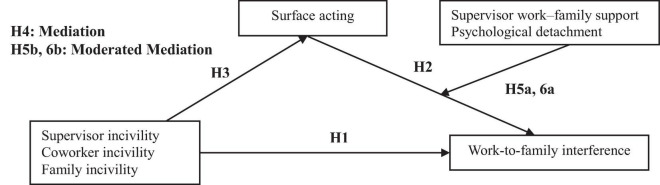
Hypothesized model.

Overall, the current research tested the effects of supervisor incivility, coworker incivility, and family Incivility on WFI in a comprehensive moderated mediation model, which posits surface acting as a mediator between incivility and WFI and both SWFS and detachment as buffers to the mediation effects (see Figure 1).

## Method

### Participants

Participants were recruited from 30 preschools located in a central province of China. Paper-and-pencil survey packets including an introduction letter and survey materials were distributed to preschool teachers, and 577 preschool teachers returned completed paper-and-pencil surveys. A total of 509 valid data were ultimately acquired (effective rate of 88.2%). Among them, 16 (3.1%) teachers were men, 490 (96.3%) were women, and whereas 3 teachers did not report their sex. A mean age of teachers was 31.03 years, spanning from 18 to 54 years. As for marital status, 206 (40.5%) were single, 301 (59.1%) were married, and 2 teachers were with unidentified marital status. With regard to children, 226 (44.4%) teachers had children living with them.

### Measures

#### Workplace incivility

The Incivility Scale (Cortina et al., 2001) was applied to measure incivility. Supervisor- and coworker-initiated incivility are each composed of seven items. Family incivility was assessed with a modified version by rephrasing wording that referred to superiors/coworkers with wording that referred to child’s family member. In addition, we dropped one item related specifically to the work situation (“Ignored or excluded you from professional camaraderie”). The 5-point rating scale (1 = never, 5 = most of the time) was utilized. An example item reads “put you down or was condescending to you.”

#### Surface acting

The Emotional Labor Scale (Brotheridge and Lee, 2003) was applied to assess surface acting. The scale consists of three items (e.g., “How often do you pretend to have emotions that you don’t really have?”). The 5-point rating scale (1 = never, 5 = always) was utilized.

#### Supervisor work–family support

The Family Supportive Supervisor Behaviors scale (Hammer et al., 2013) was applied to assess SWFS. The scale consists of four items (e.g., “Your supervisor makes you feel comfortable talking to him/her about your conflicts between work and non-work”). The 5-point rating scale (1 = strongly disagree, 5 = strongly agree) was utilized.

#### Psychological detachment

The Recovery Experience Questionnaire (Sonnentag and Fritz, 2007) was applied to measure detachment. The scale consists of four items (e.g., “I don’t think about work at all”). The 5-point rating scale (1 = strongly disagree, 5 = strongly agree) was utilized.

#### Work-to-family interference

Measure of WFI developed by Netemeyer et al. (1996), and it consisted of 5 items (e.g., “My job produces strain that makes it difficult to fulfill family duties”). The 5-point rating scale (1 = strongly disagree, 5 = strongly agree) was utilized.

#### Demographic variables

Due to the potential influence of demographic characteristics on work-family interface variables, gender, age, marital status, and live with children were chosen as control variables. First, because women are more likely to experience WFI, this study included it as a control variable (van Daalen et al., 2006). Second, as employees age, they are given multiple roles in the family (e.g., eldercare, childcare), and in turn experience more frequent work–family interference. Finally, employees who are married and living with children are also expected to perceive greater WFI (Byron, 2005).

## Results

### Preliminary Analyses

Confirmatory factor analyses (CFA) were used to compare the fit of the 7-factor measurement model (supervisor incivility, coworker incivility, family incivility, surface acting, SWFS, detachment, and WFI) with several alternative nested models (see Table 1). The result indicted that the 7-factor model fit the data adequately (χ^2^/*df* (1137.549/573) = 1.985; TLI = 0.938; CFI = 0.944; RMSEA = 0.044) than all the alternative models.

**TABLE 1:** Results of the Confirmatory Factor Analyses.

Model	Factors	χ 2	*df*	χ^2^/*df*	TLI	CFI	RMSEA
7-factor	supervisor incivility, coworker incivility, family incivility, surface acting, SWFS, detachment, and WFI	1137.549	573	1.985	0.938	0.944	0.044
6-factor	supervisor incivility, coworker incivility, family incivility, surface acting, SWFS + detachment, and WFI	2301.081	579	3.974	0.814	0.829	0.077
5-factor	supervisor incivility, coworker incivility, family incivility, surface acting + SWFS + detachment, and WFI	2799.441	584	4.794	0.763	0.780	0.086
5-factor	supervisor incivility + coworker incivility + family incivility, surface acting, SWFS, detachment, and WFI	3898.632	584	6.676	0.646	0.671	0.106
4-factor	supervisor incivility + coworker incivility + family incivility, surface acting, SWFS + detachment, and WFI	5059.844	588	8.605	0.525	0.557	0.122
3-factor	supervisor incivility + coworker incivility + family incivility, surface acting + SWFS + detachment, and WFI	5554.231	591	9.398	0.476	0.508	0.129
1-factor	supervisor incivility + coworker incivility + family incivility + surface acting + SWFS + detachment + WFI	6896.673	594	11.611	0.338	0.375	0.145

*N* = 509. χ^2^ = chi-square discrepancy; *df*, degrees of freedom; χ^2^/*df*, relative chi-square; TLI, Tucker-Lewis index; CFI, comparative fit index; RMSEA, root-mean-square error of approximation. “+” represents combination.

Table 2 displays descriptive statistics and correlations of the variables. As noted in Table 2, incivility stemming from supervisors (*r* = 0.38, *p* < 0.01), coworkers (*r* = 0.09, *p* < 0.05), and family members (*r* = 0.31, *p* < 0.01) were positively correlated with WFI, respectively. Supervisor incivility (*r* = 0.41, *p* < 0.01), coworker incivility (*r* = 0.19, *p* < 0.01), and family incivility (*r* = 0.35, *p* < 0.01) were also positively correlated with surface acting, respectively. Surface acting, in turn, had a positive correlation with WFI (*r* = 0.36, *p* < 0.01).

**TABLE 2:** Means, standard deviations, and correlations among study variables (*N* = 509).

Variables	1	2	3	4	5	6	7	8	9	10	11
1. Gender											
2. Age	−0.02										
3. Marital status	0.04	0.71**									
4. Living with Children	−0.05	0.80**	0.74**								
5. Supervisor incivility	0.02	0.02	0.07	0.01	(0.90)						
6. Coworker incivility	−0.03	0.03	0.04	−0.04	0.27**	(0.91)					
7. Family incivility	−0.00	0.04	0.07	0.03	0.35**	0.29**	(0.87)				
8. Surface acting	−0.02	−0.00	0.04	−0.03	0.41**	0.19**	0.35**	(0.81)			
9. SWFS	0.09*	0.04	0.03	−0.01	−0.36**	−0.05	−0.22**	−0.28**	(0.89)		
10. Detachment	−0.03	0.03	−0.01	0.01	−0.29**	−0.19**	−0.26**	−0.20**	0.23**	(0.90)	
11. WFI	−0.06	0.05	0.08	0.09*	0.38**	0.09*	0.31**	0.36**	−0.37**	−0.43**	(0.86)
*M*	–	31.03	–	–	2.16	2.19	2.21	2.53	3.29	3.32	2.55
*SD*	–	8.95	–	–	0.72	0.81	0.72	0.86	0.97	0.95	0.85

Reliabilities (Cronbach’s α) are on the diagonal in parentheses.

* *p* < 0.05.

** *p* < 0.01.

### Hypothesis testing

#### Main effects results

Main effects were tested using hierarchical regression analyses. Hypothesis 1 proposes that incivility will be positively linked to WFI. Because incivility comes from multiple sources, we analyzed each source of incivility separately. The control variables were entered in Model 1. Each source of incivility was added to the Model 2. As noted in Model 2 of Table 3, incivility stemming from supervisors (β = 0.38, *p* < 0.01), coworkers (β = 0.10, *p* < 0.05), and family members (β = 0.31, *p* < 0.01) were all significantly positively associated with WFI, giving full support to Hypothesis 1.

**TABLE 3:** Hierarchical Regression Analyses Examining Surface Acting as Mediator of the Relationship Between Incivility and WFI.

Variables	Model 1		Model 2		Model 3	
	β	R/R^2^/ΔR^2^	β	R/R^2^/ΔR^2^	β	R/R^2^/ΔR^2^
**Step 1: Control variables**						
Gender	−0.06	0.12/0.01/0.01	−0.06	0.40/0.16/0.15**	−0.05	0.46/0.21/0.05**
Age	−0.07		−0.05		−0.06	
Marital status	0.05		−0.00		−0.03	
Living with children	0.10		0.13		0.16*	
**Step 2: Independent variable**						
Supervisor incivility			0.38**		0.28**	
**Step 3: Mediator variable**						
Surface acting					0.26**	
**Step 1: Control variables**						
Gender	−0.06	0.12/0.01/0.01	−0.06	0.15/0.02/0.01*	−0.05	0.39/0.15/0.13**
Age	−0.07		−0.08		−0.07	
Marital status	0.05		0.04		−0.01	
Living with children	0.10		0.13		0.17*	
**Step 2: Independent variable**						
Coworker incivility			0.10*		0.03	
**Step 3: Mediator variable**						
Surface acting					0.36**	
**Step 1: Control variables**						
Gender	−0.06	0.12/0.01/0.01	−0.06	0.33/0.11/0.09**	−0.05	0.43/0.18/0.08**
Age	−0.07		−0.07		−0.07	
Marital status	0.05		0.02		−0.02	
Living with children	0.10		0.13		0.17*	
**Step 2: Independent variable**						
Family incivility			0.31**		0.20**	
**Step 3: Mediator variable**						
Surface acting					0.30**	

* *p* < 0.05.

** *p* < 0.01.

Further, after controlling for the influence of demographics, incivility stemming from supervisors (β = 0.41, *p* < 0.01), coworkers (β = 0.18, *p* < 0.01), and family members (β = 0.35, *p* < 0.01) were all positively associated with surface acting, supporting Hypothesis 3 (not presented in Table). In addition, surface acting was positively related to WFI (β = 0.29, *p* < 0.01; see Model 2 of Table 4), supporting Hypothesis 2.

**TABLE 4:** Hierarchical Regression Analyses Examining Detachment as a Moderator of the Relationship Between Surface Acting and WFI.

Variables	Model 1		Model 2		Model 3	
	β	R/R^2^/ΔR^2^	β	R/R^2^/ΔR^2^	β	R/R^2^/ΔR^2^
**Step 1: Control variables**						
Gender	−0.06	0.12/0.01/0.01	−0.02	0.47/0.22/0.21**	−0.02	0.47/0.22/0.00
Age	−0.07		−0.04		−0.05	
Marital status	0.05		0.02		0.01	
Living with children	0.10		0.12		0.13	
**Step 2: Predictor variables**						
Surface acting			0.29**		0.29**	
SWFS			−0.29**		−0.29**	
**Step 3: Interaction term**						
Surface acting × SWFS					0.05	
**Step 1: Control variables**						
Gender	−0.06	0.12/0.01/0.01	−0.06	0.53/0.28/0.27**	−0.06	0.55/0.30/0.02**
Age	−0.07		−0.04		−0.03	
Marital status	0.05		−0.01		−0.02	
Living with children	0.10		0.14*		0.13	
**Step 2: Predictor variables**						
Surface acting			0.29**		0.29**	
Detachment			−0.37**		−0.35**	
**Step 3: Interaction term**						
Surface acting × Detachment					−0.14**	

* *p* < 0.05.

** *p* < 0.01.

#### Mediation Results

According to Hypothesis 4, the incivility–WFI relationship is mediated by surface acting. We followed the four-step procedure to test the mediation (Baron and Kenny, 1986). First, it must be shown that the effects of the independent variables (the three sources of incivility) on the dependent variable (WFI) are significant, and test of Hypothesis 1 bears this out. Second, it is necessary to demonstrate that the independent variables are related to the mediator (surface acting), and the test of Hypothesis 3 satisfies the second requirement. Third, it is necessary to show a significant relation between the mediator (surface acting) and the outcome variable (WFI), and the test of Hypothesis 2 proves the relation.

Finally, to establish partial or complete mediation, the effect of the independent variable on the dependent variable should be reduced substantially or even non-significant when entering into the mediator (surface acting). As shown in Model 3 of Table 3, the effect of coworker incivility on WFI is non-significant when surface acting enters into the model; thus, surface acting completely mediates the effect of coworker incivility on WFI. However, the effects of supervisor and family incivility on WFI remain significant when controlling for surface acting. Nevertheless, the standardized beta coefficient does decrease appreciably, from.38/.31 (*p* < 0.01) to 0.28/0.20 (*p* < 0.01), indicating that surface acting partially mediates these relationships.

Bootstrap tests were run to further verify the indirect effect and confidence intervals (CI) by using model 4 of Hayes (2013) PROCESS macro. Supervisor incivility was positively related to WFI indirectly through surface acting (Total effect = 0.4514, SE = 0.0486, 95% CI = [0.3560, 0.5469]; Direct effect = 0.3293, SE = 0.0516, 95% CI = [0.2280, 0.4306]; Indirect effect = 0.1221, SE = 0.0316, 95% CI = [0.0658, 0.1900]). Similar results were obtained for the indirect effects of coworker incivility (Total effect = 0.1052, SE = 00475, 95% CI = [0.0118, 0.1986]; Direct effect = 0.0341, SE = 0.0452, 95% CI = [−0.0546, 0.1229]; Indirect effect = 0.0711, SE = 0.0248, 95% CI = [0.0273, 0.1246]), and family incivility (Total effect = 0.3625, SE = 0.0499, 95% CI = [0.2645, 0.4606]; Direct effect = 0.2402, SE = 0.0510, 95% CI = [0.1399, 0.3404]; Indirect effect = 0.1224, SE = 0.0325, 95% CI = [0.0663, 0.1934]) on WFI. Significant indirect effect is proven because 95% CI don’t contain a zero, supporting Hypothesis 4 partially.

#### Moderation Results

**FIGURE 2:**
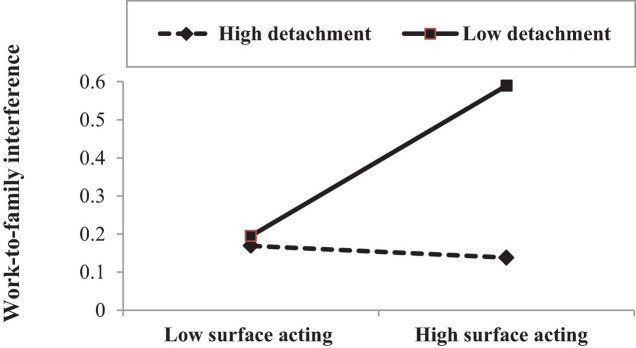
Moderating role of detachment on surface acting–work-to-family interference relationship.

Moderated regression analyses were used to test the interactive effects. We added controls in Model 1 (i.e., demographics) and entered predictor and moderator (i.e., surface acting and SWFS/detachment) and the interaction term in Model 2 and Model 3, respectively. Prior to the analyses, both predictor and moderator were mean-centered in order to reduce multicolinearity (Aiken and West, 1991). As noted in Table 4, detachment significantly alleviates the effect of surface acting (β = −0.14, *p* < 0.01) on WFI. This significant interaction was plotted according to Aiken and West (1991) methods. As shown in Figure 2, the effect of surface acting on WFI becomes weaker at higher detachment and stronger at lower detachment, supporting Hypothesis 6a. The moderating effect of SWFS was not significant and Hypothesis 5a was not supported.

#### Moderated Mediation Results

Conditional indirect effects were tested using Model 14 of Hayes (2013) PROCESS macro (5,000 bootstrapped samples and 95% CI). As noted in Table 5, the mediating effect of surface acting changed depending on the level of detachment and was weakest at lower (− 1 SD) level of it. Specifically, the effect of supervisor incivility on WFI through surface acting was significant at low (Effect = 0.1703, SE = 0.0359, 95% CI = [0.1079, 0.2480]), rather than high (Effect = 0.0232, SE = 0.0371, 95% CI = [−0.0405, 0.1052]) levels of detachment. Similarly, the effect of family incivility on WFI through surface acting was significant at low (Effect = 0.1535, SE = 0.0346, 95% CI = [0.0930, 0.2296]), rather than high (Effect = 0.0507, SE = 0.0331, 95% CI = [−0.0001, 0.1269]) levels of detachment. However, the effect of coworker incivility on WFI through surface acting was significant (Effect = 0.0841, SE = 0.0288, 95% CI = [0.0336, 0.1468]) when detachment was low, whereas the effect was still significant but reduced (Effect = 0.0274, SE = 0.0182, 95% CI = [0.0018, 0.0709]) when detachment was high. The calculation of the index of moderated mediation confirms a true conditional indirect impact because 95% CI did not overlap with zero for any of the models (Hayes, 2015; Hayes et al., 2017). These results provide support for Hypothesis 6b.

**TABLE 5:** Analysis of Conditional Indirect Effects of Surface Acting at Various Values of Detachment.

Values of Detachment	Conditional indirect effect	*SE*	Lower CI	Upper CI
**Independent variable: Supervisor Incivility**				
– 1 SD	0.1703	0.0359	0.1079	0.2480
M	0.0886	0.0270	0.0411	0.1486
+ 1 SD	0.0232	0.0371	−0.0405	0.1052
Index of moderated mediation	−0.0654	0.0221	−0.1094	−0.0213
**Independent variable: Coworker Incivility**				
− 1 SD	0.0841	0.0288	0.0336	0.1468
M	0.0526	0.0197	0.0195	0.0962
+ 1 SD	0.0274	0.0182	0.0018	0.0709
Index of moderated mediation	−0.0252	0.0114	−0.0508	−0.0061
**Independent variable: Family Incivility**				
− 1 SD	0.1535	0.0346	0.0930	0.2296
M	0.0964	0.0278	0.0507	0.1591
+ 1 SD	0.0507	0.0331	−0.0001	0.1269
Index of moderated mediation	−0.0457	0.0172	−0.0796	−0.0114

Bootstrap sample size = 5,000. CI, confidence interval.

## Discussion

Adhering to the tenet of COR theory and consistent with the need for occupation-specific investigations to disclose whether, why, and when incivility from different sources influence WFI, the current study examined the relative contribution of incivility from supervisors and coworkers as well as a unique source of incivility to preschool teachers, namely family incivility. We found that each of the three sources of incivility was related to WFI; however, supervisor incivility exhibited more detrimental impacts compared with coworker and family incivility. We also found support for a mediating mechanism in which incivility stemming from supervisors, coworkers, and family members are related to WFI, and surface acting mediated these relationships. Regarding the moderating role of SWFS and detachment, no significant interaction effect was found between surface acting and SWFS on WFI, while detachment did fulfill a buffering role in the surface acting–WFI relationship. Furthermore, we demonstrated a moderated mediation model in which the relationship between incivility and WFI via surface acting was weaker for teachers experiencing higher levels of detachment after work. Taking the results together, these results support application of the principles of COR theory, and extend prior research by determining whether, why, and when workplace incivility has implications for preschool teachers’ family lives.

### Theoretical Implications

This study focused on the under-researched area of handling incivility in the work–family literature, and thus yielded some important theoretical contributions. First, the present research is among the first to explore whether the detrimental effects of incivility can spill over to employees’ family lives, increasing WFI. The positive relationships of incivility from supervisors, coworkers, and family members with experience of WFI suggest that incivility tends to consume individuals’ valued resources (e.g., emotional, cognitive, and social-capital resources), leaving them with fewer resources to efficiently deal with family needs and responsibilities and thus experience more WFI. This consistent positive connection between incivility stemming from multiple sources and WFI helps confirm the stability of the incivility-WFI relationship. In addition, despite the widespread belief that incivility stemming from different sources can have different effects, the empirical evidence is scarce and inconclusive. Further analysis found that employees facing supervisor incivility tend to perform high levels of WFI in comparison with those facing incivility from coworkers and family members. At least in theory, this is due to the high power distance between leaders and employees that makes the experience of supervisor incivility most outstanding and likely results in more serious resource loss; by contrast, incivility from coworkers and family members might not be assessed as stressful as supervisor incivility and thus will not threaten their personal resources seriously (e.g., Caza and Cortina, 2007; Hershcovis et al., 2017). The differential relationships between each source of incivility and WFI also highlight the importance of containing multiple sources of incivility to better view the complexity of preschool teachers’ interpersonal relationships as well as the whole social environment of the workplace.

Second, the second major contribution of our research stems from confirming the role of incivility in eliciting surface acting very clearly. Our findings indicated that incivility is related to surface acting, which was consistent with other research studying interpersonal stressors as the predictors of emotional labor strategies (Carlson et al., 2012; Grandey et al., 2012; Adams and Webster, 2013). There are two possible reasons for this: on the one hand, in the face of incivility, preschool teachers perform surface acting by faking, amplifying, or suppressing felt emotions to exhibit what is expected, to meet the requirements of emotional display rules. On the other hand, when preschool teachers experience incivility, they may display surface acting in order to avoid losing cognitive and emotional resources that they strive to protect, retain, and build (Hobfoll, 1989). Furthermore, results show that employees facing incivility arising from interactions with supervisors are likely to report more surface acting. Drawing upon the COR theory (Hobfoll, 2001), employees may highly regulate their emotions when dealing with supervisor incivility for the sake of impression management by making a good impression on supervisors because of the need of protecting such resources.

Third, our third theoretical contribution lies in examining surface acting as a mediator that help clarify the incivility-family relationship. Although both incivility and emotion labor have been proven to have unique impacts on WFI, we theoretically integrate incivility and emotional labor literature in one model and found that incivility and surface acting work together to create a resource depletion mechanism that greatly aggravates WFI. In the process, incivility first produces the initial resource loss, and surface acting as an imperfect coping response that further exacerbates the loss of resources, which ultimately increase WFI. Our results are consistent with prior research that surface acting mediated the relationships of other forms of workplace mistreatment such as abusive supervision with WFI (Carlson et al., 2012). Integrating emotional labor into the incivility literature provides a promising idea for understanding not only why, but also how workplace incivility negatively impacts the quality of employees’ personal lives.

Finally, our study extends knowledge about the type of moderators that are likely to reduce the resource losses due to coping with incivility and subsequent surface acting. Specifically, we highlight the importance of SWFS and detachment as effective situational and personal resources for buffering against the resource consumption of surface acting when coping with incivility, thereby reducing the spillover of incivility into the nonwork domain. We found that detachment indeed combats the harmful impacts of surface acting originated from incivility. Preschool teachers who are able to successfully detach from their work reported less WFI when engaging in surface acting. The result is generally consistent with previous findings that detachment serves as a buffer against the harmful impacts of work–family relationships (Demsky et al., 2019; Gu and Wang, 2021). The moderation effects of detachment confirm the importance of investigating boundary conditions of incivility’s effects on the basis of the recovery mechanism. Moreover, we further validate a moderated mediation model in which the indirect impact of incivility on WFI via surface acting became weaker for employees perceived more detachment. In other words, the more an individual perceived having detachment, the less impact surface acting had on the individuals’ family when the individual experienced incivility. Our study addresses the buffering effects of detachment on the indirect relationship between incivility and WFI, which, to our knowledge has not been examined previously. This synthesis model can simultaneously answer why (mediation) and when (moderation) incivility has a deleterious effect on employees’ family lives.

### Practical implications

In light of our findings, designing interventions to reduce negative effects of incivility on employees’ family lives should consider the source of the incivility, mediators and moderators. First, direct efforts aimed at the prevention of incivility are important. Thus, the most fundamental thing for an organization is to implement relevant policies, programs, and practices that can best cultivate a civil and respect work climate, such as providing interpersonal training by encouraging respectful workplace interactions or fostering an environment of inclusion by regular team-building activities. In addition, although the results do indicate that incivility stemming from supervisors, coworkers, and family members all have a significant deleterious impact on employees’ family lives, the influence weights of these sources are not the same. Therefore, organizations and mangers are advised to differentiate between these different sources and focus more of their attention on supervisor incivility.

Second, although it is necessary to prevent the occurrence of incivility, it is also important to prevent and address the downstream negative effects of incivility. The mediating effect of surface acting suggests that it is practicable to provide an interruption to help employees manage and reduce surface acting. This is particularly important for employees who often frequently suppressing and faking their emotions when facing negative interpersonal interactions. Organizations could also implement practices to value and compensate emotional work with financial rewards or provide social support (Grandey et al., 2013). In addition to making an effort to ameliorate the depleting effects of surface acting, it is more effective to provide training programs that help employees to adopt more effective emotional labor strategies such as deep acting to respond to incivility (Hülsheger and Schewe, 2011). Training employees on how to engage in deep acting could include trained emotional intelligence, such as understanding other people’s emotions and dealing with them effectively, and trained imagination, such as thinking of positive interactions with supervisors and family members (Hochschild, 1983).

Finally, our findings indicated that detachment acts as a critical buffer through which employees feel less WFI when engaging in surface acting. Thus, creating positive conditions and favorable atmosphere for employees to effectively detach from work is crucial here. Previous studies have suggested that combined interventions integrating work-directed strategies, such as setting flexible work schedules, modest work breaks, and workshops about time management, and person-directed strategies, such as positive work reflection, mindfulness, boundary management (e.g., goal-setting techniques separating the work and home spheres), and taking part in meaningful leisure activities (e.g., yoga, Tai Ji) during nonwork time, would boost employees’ detachment skills (Hahn et al., 2011; Hülsheger et al., 2014; Karabinski et al., 2021).

### Limitations, suggestions, and conclusions

Despite its contributions, there are some limitations for our research. First, this research used a self-report questionnaire, which may have common-method bias. Future studies could collect more objective data from multiple sources, such as coworker- or supervisor-report measure of surface acting and spouse rating for detachment. Second, the design was cross-sectional that limits on inferences surrounding causality. Diary, longitudinal, or even experimental data can be used to confirm the potential causal linkages between incivility and WFI. Third, as we sampled only preschool teachers only in a province and 59.1% teachers were married (44.4% had children living with them), it inevitably raises concerns about the generalizability of our findings. It would be necessary to examine whether the findings are stable across different regional cultures and occupational groups. Lastly, we had limited our scope to explore the two resource-providing variables (SWFS and detachment) only. It might be valuable to explore other resource-providing variables.

In conclusion, this study is among the first to investigate to test the mediating and moderating mechanisms underlying the incivility-WFI relationship. By drawing on the principles of COR theory, this research provides empirical evidence for the excessive resource consumption following incivility, via surface acting. We found that surface acting can serve as one potential mechanism by which incivility is related to WFI. Moreover, the mediation mechanism was moderated by detachment such that the adverse impact of incivility on WFI via surface acting is weaker for preschool teachers with higher levels of detachment. Our results suggest that boosting detachment from work is crucial to sustaining a balance between work and family. Moderated mediation model enrich an understanding of how and when incivility negatively influence employees’ family lives.

## Data availability statement

The original contributions presented in the study are included in the article/supplementary material, further inquiries can be directed to the corresponding author.

## Ethics statement

Ethical review and approval was not required for the study on human participants in accordance with the local legislation and institutional requirements. This study complies with the current laws in China. The studies were conducted in accordance with the local legislation and institutional requirements. The participants provided their written informed consent to participate in this study.

## Author contributions

YG: Conceptualization, Formal analysis, Funding acquisition, Methodology, Project administration, Validation, Writing−original draft. CW: Formal analysis, Funding acquisition, Investigation, Methodology, Writing−review and editing. JM: Funding acquisition, Writing−review and editing, Conceptualization, Resources, Supervision.
